# Tensile Fatigue Behavior of Polyester and Vinyl Ester Based GFRP Laminates—A Comparative Evaluation

**DOI:** 10.3390/polym13030386

**Published:** 2021-01-27

**Authors:** Wahid Ferdous, Allan Manalo, Peng Yu, Choman Salih, Rajab Abousnina, Tom Heyer, Peter Schubel

**Affiliations:** 1 Centre for Future Materials (CFM), University of Southern Queensland, Toowoomba, QLD 4350, Australia; Allan.Manalo@usq.edu.au (A.M.); Peng.Yu@usq.edu.au (P.Y.); Choman.Salih@usq.edu.au (C.S.); Rajab.Abousnina@usq.edu.au (R.A.); Peter.Schubel@usq.edu.au (P.S.); 2 Austrak Pty. Ltd., Brisbane, QLD 4001, Australia; Tom.Heyer@vossloh.com

**Keywords:** fatigue, fibre composites, polyester and vinyl ester resins, stress ratio, fatigue model

## Abstract

Fatigue loading is critical to fibre reinforced polymer composites due to their anisotropic and heterogenous nature. This study investigated the tensile fatigue behaviour of polyester and vinyl ester based GFRP laminates to understand the critical aspects of failure mode and fatigue life under cyclic loading. GFRP laminates with two different resin systems (polyester and vinyl ester), two different stress ratios (0.1 and 0.5) and two different environmental conditions (air and water) were investigated at an applied stress of 80%, 60%, and 40% of the ultimate capacity. Based on the investigated parameters (i.e., resin types, stress ratio, environmental conditioning, and maximum applied stress), a fatigue model was proposed. Results show that the resin system plays a great role in fatigue failure mode while the stress ratio and environmental condition significantly affect the tensile fatigue life of GFRP laminates. The types of resin used in GFRP laminates and environmental conditions as input parameters in the proposed fatigue model are a unique contribution.

## 1. Introduction

The behaviour of fibre reinforced polymer (FRP) composite laminates under cyclic loading are found to be different and more critical than the conventional isotropic materials due to non-uniform stress distribution [1,2]. FRP composites are currently being used in marine and civil infrastructure applications due to their corrosion resistance and lightweight [3,4,5,6,7,8,9,10]. These structures are often subjected to cyclic loading from different sources. So far, the behaviour of fibre composites are well studied in static loading condition [11,12]; however, the investigations are still very limited for dynamic fatigue loading. This study aimed to investigate fatigue behaviour of glass fibre reinforced polymer (GFRP) composites to understand the critical aspects of failure mode and fatigue life.

There are several resin systems that are commercially available in manufacturing composites. The two common types are polyester and vinyl ester resins. Polyester is the preferred choice when cost is an important factor [13]. On the other hand, the static mechanical performance of vinyl ester based composites is better than polyester composites [14,15]. However, it is still unclear how polyester and vinyl ester based GFRP composites will behave under cyclic loading. This study evaluated the comparative performance of polyester and vinyl ester based GFRP laminates under cyclic loading.

The stress amplitude is an important parameter for fatigue life of a material. Keller et al. [16] studied the tensile fatigue behaviour of pultruded GFRP profiles for the stress amplitude of 0.1. Vieira et al. [17] also investigated the fatigue behaviour of pultruded GFRP composites at a stress ratio of 0.1. Borrego et al. [18] studied a very low stress ratio (0.05) to investigate the effectiveness of nanoparticles to improve fatigue life of GFRP composites. Similarly, most of the previous studies are conducted fatigue investigation under a constant stress ratio. However, the load cycles in a structural component vary its magnitude. For example, the bridge girders are subjected to load from vehicles having different axle loads. Therefore, it is important to investigate how the stress ratio affect fatigue life. This study investigated the effect of stress ratio on the fatigue life of GFRP composites.

The outdoor structures are frequently subjected to moisture, rain, humidity, and thermal alterations [19]. These extreme environments were found to be a critical parameter that can deteriorate the properties of materials [20]. Benmokrane et al. [13] found that the vinyl ester based composites have superior resistance in moisture environment than polyester based composites due to the lower degradation at fiber–resin interface. Liew and Tan [21] studied the performance of GFRP composites under tropical climate and found that the strength of GFRP laminates was decreased with exposure time. Aboelseoud and Myers [22] investigated the effect of five different environmental conditions on the performance of hybrid composite beam. They concluded that the chemical reaction caused by the weathering action can increase the chances of fiber-matrix debonding. Most of these studies were focused on the static performance of laminated composites under extreme weather conditions. However, it is not fully understood how the extreme environment can affect the fatigue life of GFRP composites. The present study highlighted the effect of water absorption on the fatigue life of GFRP composites. It is expected that the outcome of this study will benefit researchers and design engineers with improved understanding of the fatigue behaviour of GFRP composites.

## 2. Materials and Methods

### 2.1. Materials

Two different resin systems such as polyester and vinyl ester were used to fabricate the laminates using hand lay-up manufacturing process and cured 24 h at 60 °C in a temperature controlled room. These two resin systems were selected based on the performance and cost criteria. While vinyl ester has superior mechanical and durability properties; however, it is more expensive than polyester resin. This study provided an opportunity to understand the fatigue behaviour of composite laminates made of these two resin systems. The fibres in the GFRP laminates were oriented in both longitudinal and transverse directions with the same fibre orientations for both types of laminate. The fibre volume ratio was 55% with a laminate density of 2000 kg/m^3^ for both laminates in accordance with ASTM D3171 [23]. The laminated specimens were cut from the composite plates at a nominal dimension of 300 mm (L) × 25 mm (W) × 3.5 mm (T). Both ends of the sample were glued (techniglue R60 resin and H60F hardener, structural epoxy adhesive) with 50 mm tabs that leave the specimens’ gauge length of 200 mm.

### 2.2. Justification of Selecting Parameters

This study investigated three different parameters including types of resin, applied stress ratio and environmental conditions. Since polyester and vinyl ester are two of the most commonly used resin systems, it is worth investigating their behaviour under fatigue load. Fatigue loads on engineering strcutures are greatly varying in magnitude. For example, a number of vehicles having different axle loads are running over the bridge that creating fatigue at different load levels. The applied stress ratios (minimum-to-maximum applied stress) of 0.1 and 0.5 were considered to represent this loading scenario on the structures. Moreover, the outdoor structures are often subjected to rain and moisture which may affect the fatigue life of exterior composite laminates. To address this environmental condition, the specimens were submerged under water for one month before testing them in fatigue. All these samples were tested at an applied stress of 80%, 60%, and 40% of the ultimate capacity. Table 1 provides a list of parameters investigated in this study. The sample name R_P_R_0.1_E_N_S_80_ represents that a polyester resin (R_P_) based sample was subjected to 0.1 stress ratio (R_0.1_) and cured at normal environmental condition (E_N_) before applied 80% stress (S_80_) of the ultimate capacity.

### 2.3. Test Setup

Five replicate samples were tested (static) at a loading rate of 2 mm/min to determine the ultimate capacity and modulus of elasticity of the laminates in accordance with ASTM-D3039 [24]. Tension–tension fatigue test was performed based on ISO-13003 standard [25]. Both tests were conducted with a computer-controlled servo-hydraulic MTS having a capacity of 100 kN. To prevent the premature failure due to slipping at the gripping area, the specimens were clamped carefully with sufficient pressure applied onto the wedge jaws. All tests were performed at a room temperature of 23 °C and relative humidity of 50%. The stress ratios (R) were kept positive for all tests to create a tension-tension fatigue loading scenario. The number of cycles, load, and displacement data were recorded at regular intervals. Three replicate samples for 80% and 60% and two samples for 40% of the ultimate load were tested to obtain higher accuracy in fatigue results. The specimens at 80%, 60%, and 40% of the ultimate load were tested at 2, 4, and 6 Hz frequencies, respectively. Since the specimen requires a high number of cycles to fail at 40% of the ultimate load, the number of replicate samples were reduced to two.

**Figure 1:**
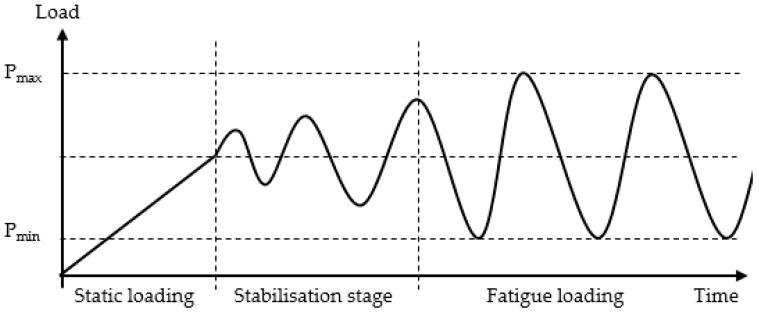
Fatigue load transmission.

A constant amplitude of load was applied at force control mode with a sinusoidal waveform. The static load transmitted into the specimen followed by variable amplitude of sinusoidal load (stabilization stage, within approximately 1 s), which was happened within a very short period of time at the beginning. Once the load stabilised, it continues with constant amplitude until the failure of the specimen. The loading configuration is graphically shown in Figure 1.

## 3. Results and Discussion

### 3.1. Ultimate Strength and Stiffness

**Figure 2:**
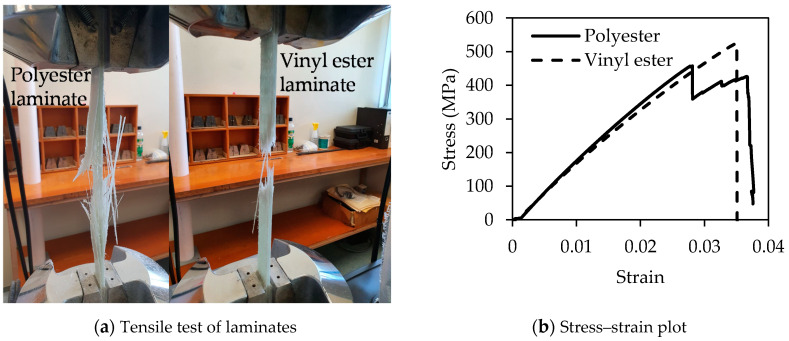
Ultimate test of polyester and vinyl ester based GFRP laminates.

The static test was conducted for both polyester and vinyl ester based GFRP laminates to determine the ultimate capacity and modulus of elasticity. This test is particularly important to determine the applied stress level for the fatigue test. The representative failure of both laminates are shown in Figure 2a. It can be seen that the nature of failure for polyester and vinyl ester laminates are different. Polyester samples failed in explosive manner at the middle of the gauge in two stages. The majority of the outer fibres were failed first and a drop of stress was noticed in the stress–strain curve (Figure 2b). Thereafter, the inner fibres started to carry the load and the stress again started to increase gradually. An ultimate failure was observed when inner fibres were failed. On the other hand, the vinyl ester based laminates were failed laterally at the middle of the gauge in a brittle manner. This result has supported the findings of Boinard et al. [26] where they indicated the fibre-matrix bond is stronger in vinyl ester based laminates than polyester. The average strength and modulus of elasticity of polyester based laminates were 484 MPa (standard deviation 19.6) and 20 GPa (standard deviation 1.4), respectively while these values were 524 MPa (standard deviation 28.2) and 19.5 GPa (standard deviation 0.7) for vinyl ester based laminates.

### 3.2. Fatigue Failure Mode

**Figure 3:**
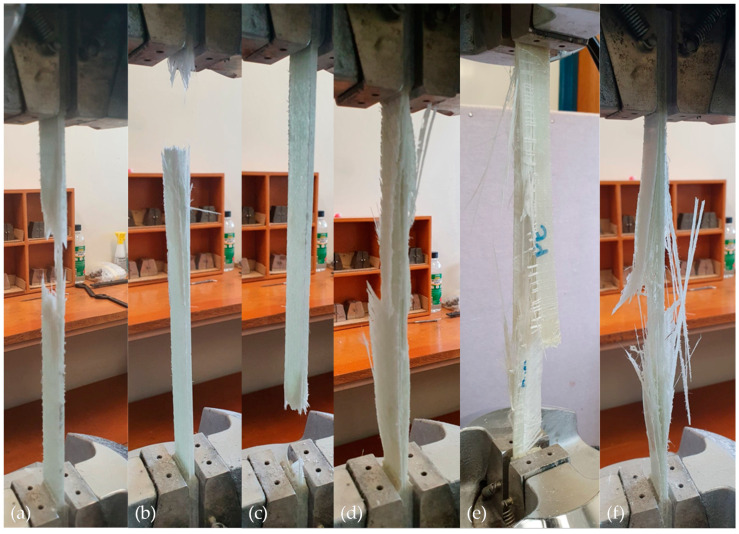
Failure of the specimens recorded during the test. (**a**) Lateral failure at the middle of the gauge, (**b**) lateral failure at the top tab, (**c**) lateral failure at the bottom tab, (**d**) edge delamination at the middle of the gauge, (**e**) surface delamination at the middle of the gauge, and (**f**) explosive failure at the middle of the gauge.

The fatigue life of FRP laminates is very much dependent on their nature of the failure. Six different modes of failure such as lateral failure at the middle of the gauge (Figure 3a), lateral failure at the top tab (Figure 3b), lateral failure at the bottom tab (Figure 3c), edge delamination at the middle of the gauge (Figure 3d), surface delamination at the middle of the gauge (Figure 3e), and explosive failure at the middle of the gauge (Figure 3f) were recorded during the test. These modes of failures are expected for the laminates according to ASTM D3039 [24]. The failure obtained in Figure 3a,f are the desirable modes of failure under tension-tension cycle loading as they are failed in pure tension. The failure near the tab area (Figure 3b,c) is occurred due to stress concentration while the interlaminar delamination played a major role for the failure presented in Figure 3d,e. To avoid the failure at the tab, it is recommended to consider several factors such as tab alignment, tab angle, tab material, tab adhesive, grip pressure, grip type, and grip alignment.

### 3.3. Effect of Resin Types

**Figure 4:**
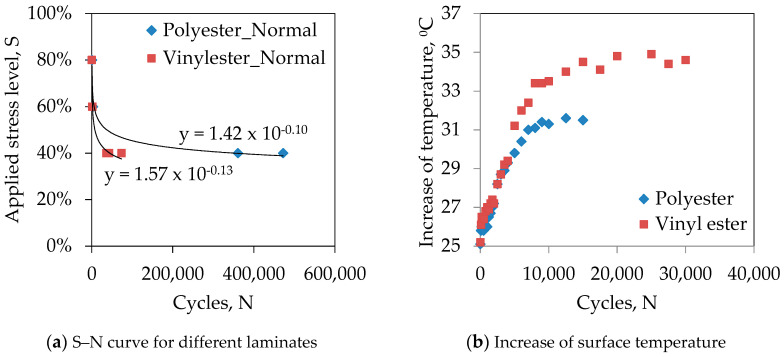
Comparison between polyester and vinyl ester based laminates.

Polyester and vinyl ester are widely used resin systems in polymer composites due to their good balance between performance and cost. Understanding the fatigue behaviour of laminates made of these two resin systems is important for designing structures subjected to cyclic loading. Figure 4a plotted the S–N (stress vs. cycle) curves for polyester and vinyl ester based GFRP laminates at the normal environmental condition. It can be seen that the polyester based samples were failed in higher cycles than vinyl ester based samples at the same stress level, particularly at 40%. This can be attributed to the mode of failures of the samples and generation of temperature during cyclic loading. Specimen R_P_R_0.1_E_N_S_40_ failed in explosive manner at the middle of the gauge (Figure 3f) while R_V_R_0.1_E_N_S_40_ failed laterally at the top tab (Figure 3b). The interaction between secondary hydroxyl groups in the vinyl ester molecule and the hydroxyl groups present on the surface of glass fibre can improve bonding of the resin to the fibres (i.e., rigid bond) [26]. The premature failure of R_V_R_0.1_E_N_S_40_ specimen perhaps due to the rigid bond that created stress concentration and provided lower fatigue life than R_P_R_0.1_E_N_S_40_. This result is in-line with the findings of Ferdous et al. [27] where it was shown that the premature failure has a significant impact on fatigue life of laminates. Moreover, the generation of surface temperature measured by digital infrared thermometer during cyclic loading was shown that the vinyl ester resin system generates more temperature than polyester resin. Figure 4b plotted the variation of the surface temperature of polyester and vinyl ester based GFRP laminates which show that the temperature increases up to 6.5 °C for polyester and 10 °C for vinyl ester based laminates. The surface temperature increased rapidly for the first 10,000 cycles due to the internal friction between particles and then increase slowly due to the gradual reduction of internal friction. In general, the rigid bond of vinyl ester resin system than polyester makes the GFRP laminates more prone to stress concentration under cyclic loading.

### 3.4. Effect of Stress Ratio

**Figure 5:**
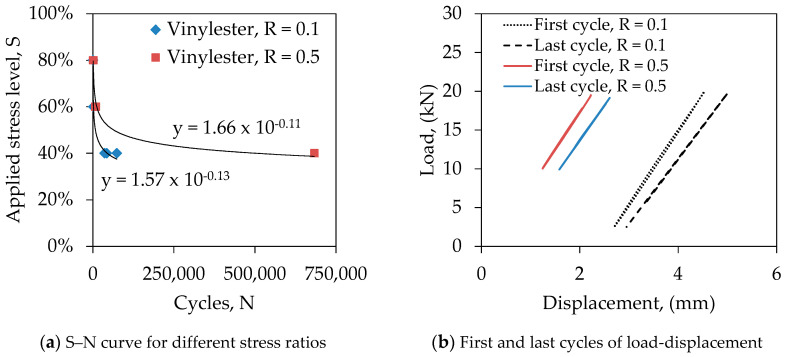
Comparison between different stress ratios.

Structures are subjected to different level of cyclic loading such as low to medium and low to high range of loads on which the fatigue life of structures are dependent. This effect can be captured by varying the stress ratio during a fatigue test. Figure 5a represents the S–N curve of GFRP laminates at a stress ratio of 0.1 and 0.5. It can be seen that the fatigue life of laminate is significantly affected by the stress ratio. At an applied stress of 40%, the fatigue life increased by almost 10 times for a stress ratio of 0.5 compared to 0.1. This can be explained by the loss of stiffness of laminates between first and last cycles [27]. Figure 5b plotted the load–displacement curve of laminates for first and last cycles at a stress ratio of 0.1 and 0.5. The slope of the load–displacement curve is the function of stiffness. It can be seen that the specimen R_V_R_0.1_E_N_S_40_ lost 11.32% stiffness while the specimen R_V_R_0.5_E_N_S_40_ lost only 7.03% stiffness before the failure. The higher loss of stiffness reduced fatigue life. The lower stress ratio means the higher elongation of laminates in each cycle which increase the surface temperature due to higher internal friction and degrade the fatigue resistance quicker than what happen for higher stress ratio. This implies the higher stress ratio is less detrimental to structures under cyclic loading than lower stress ratio.

### 3.5. Effect of Water Absorption

**Figure 6:**
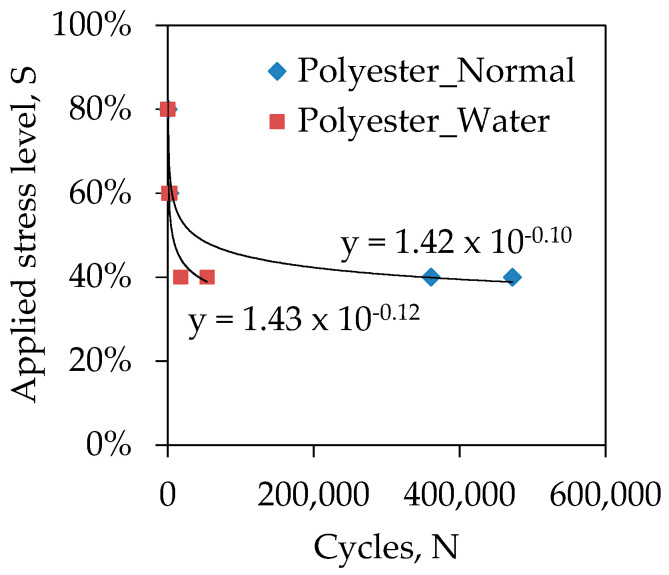
Comparison between different environmental conditions.

Outdoor structures often come into contact with water from rain, moisture, or other sources. This may allow structures to absorb water which may affect their fatigue life. Therefore, a comparative study was conducted to understand the effect of water absorption on the fatigue life of GFRP laminates with respect to the normal environmental condition. Before fatigue testing, samples were submerged into water for one month (30 days) to allow sufficient absorption of water. Quino et al. [28] found that the water absorption in GFRP laminates can reach to the saturation level after approximately 600 h (25 days) meaning that the water absorbtion was reached to an equilibrium condition. It was found that the GFRP laminates absorbed 0.38% water with respect to the initial weight of the samples. This small amount of water affected the fatigue life significantly as can be seen from Figure 6 that plotted the S–N curve of GFRP laminates cured at normal air and water environments. The fatigue life of water samples decreased to one-ninth of the normal samples when the specimens were subjected to 40% of the ultimate strength of normal samples. This can be attributed to the change of fatigue failure mode when absorbed water. The normal specimen R_P_R_0.1_E_N_S_40_ was failed in explosive manner at the middle of the gauge (Figure 3f) while the specimen R_P_R_0.1_E_W_S_40_ ageing in water was failed in delamination (Figure 3e). Boinard et al. [26] found that the rate of water absorption of polyester based laminates is twice than vinyl ester based laminates. The hydrolysis of the matrix and fibre, and swelling of the matrix due to the loss of physical interactions destabilise the fibre–matrix interface that reduce the modulus of matrix material and decrease the transverse flexural strength and results a premature failure of the specimens.

## 4. Fatigue Model

**Figure 7:**
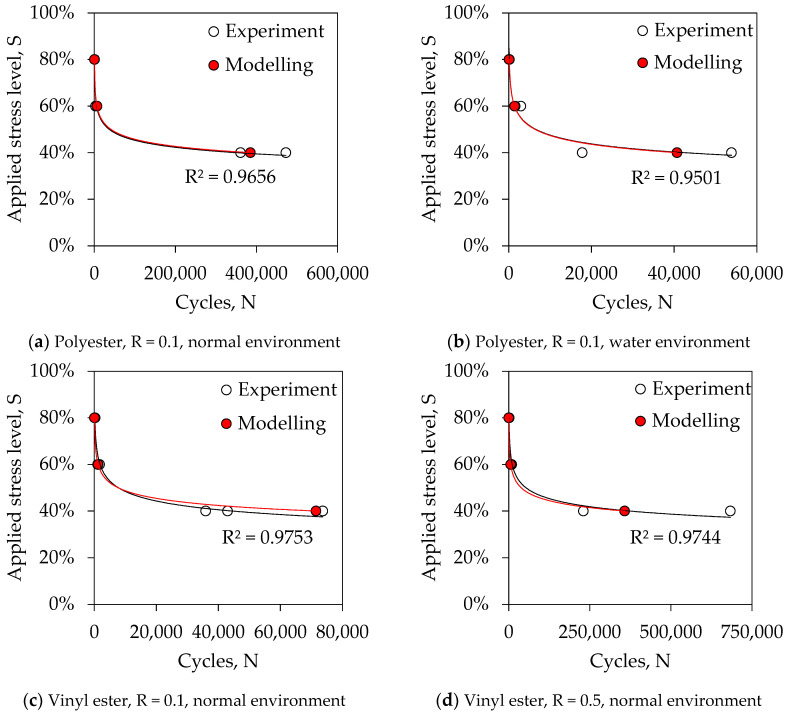
Comparison between results from experiments and proposed model.

This study investigated twelve different configurations of samples including two resin systems, two stress ratios and two environmental conditions at three load levels as tabulated in Table 1. It is desirable to capture the fatigue behaviour of GFRP laminates by a single equation incorporating all variables. This study proposed a fatigue model (Equation (1)) that is applicable to all twelve samples. This model was developed based on the variation of results observed in Figure 4a, Figure 5a and Figure 6. It is assumed that the variation of test frequency has no effect on the fatigue life of laminates. In Equation (1), *N* represents the fatigue life, *R* is the stress ratio, *S* is the maximum applied stress, *a* is the resin constant and *b* is the environmental constant. It was found that the resin constant for polyester and vinyl ester resin systems are 7 and 1.3, respectively. On the other hand, the environmental constant for normal curing is 1 while this magnitude is reduced to 0.83 for samples submerged in water. Figure 7 plotted the variation of model results from the experiments. It can be seen that the model can satisfactorily predict the experimental behaviour.

(1) $$N=aR{\left(\frac{1.5}{S}\right)}^{10b}$$

## 5. Conclusions

The tensile fatigue behaviour of polyester and vinyl ester based GFRP laminates are compared. The effect of different types of resin, applied stress ratio and environmental conditions are investigated experimentally and a fatigue model is developed. Based on the results, the following conclusions are drawn:

Polyester resin based GFRP laminates primarily failed in explosive manner at the middle of the gauge while the laminates composed of vinyl ester resin are failed laterally under cyclic loading. The absorption of water can change the nature of fatigue failure to delamination.

Vinyl ester resin system can create a rigid bond with fibres and generate more heat than polyester resin. The rigid bond of the vinyl ester resin system than polyester makes the GFRP laminates more prone to stress concentration under cyclic loading.

The stress ratio can play a major role in fatigue life of composite structures. The higher stress ratio is less detrimental to structures under cyclic loading than lower stress ratio. This is due to the lower loss of stiffness at higher stress ratio.

The absorption of water makes the bond between fibres and matrix of GFRP laminates weaker. This weak bond force the specimen to fail prematurely and significantly impact the fatigue life.

A simplified fatigue model is proposed by considering the types of resin and curing environments as a function of fatigue life. The proposed model well captured the fatigue behaviour for all resin systems, stress ratios, environmental conditions, and applied stress levels investigated in this study.

## Figures and Tables

**Table 1:** Parameters investigated in this study.

Sample Name	Types of Resin	Stress Ratio, R	Environmental Conditions	Applied Max Stress Level, S	No. of Tested Samples	Failure Modes
R_P_R_0.1_E_N_S_80_	Polyester	0.1	Normal	80%	3	Figure 3b
R_P_R_0.1_E_N_S_60_	Polyester	0.1	Normal	60%	3	Figure 3b
R_P_R_0.1_E_N_S_40_	Polyester	0.1	Normal	40%	2	Figure 3f
R_P_R_0.1_E_W_S_80_	Polyester	0.1	Water	80%	3	Figure 3d
R_P_R_0.1_E_W_S_60_	Polyester	0.1	Water	60%	3	Figure 3d
R_P_R_0.1_E_W_S_40_	Polyester	0.1	Water	40%	2	Figure 3e
R_V_R_0.1_E_N_S_80_	Vinyl ester	0.1	Normal	80%	3	Figure 3c
R_V_R_0.1_E_N_S_60_	Vinyl ester	0.1	Normal	60%	3	Figure 3c
R_V_R_0.1_E_N_S_40_	Vinyl ester	0.1	Normal	40%	2	Figure 3b
R_V_R_0.5_E_N_S_80_	Vinyl ester	0.5	Normal	80%	3	Figure 3c
R_V_R_0.5_E_N_S_60_	Vinyl ester	0.5	Normal	60%	3	Figure 3c
R_V_R_0.5_E_N_S_40_	Vinyl ester	0.5	Normal	40%	2	Figure 3a

## Data Availability

Not applicable.
